# Levo-Tetrahydroberberrubine Produces Anxiolytic-Like Effects in Mice through the 5-HT_*1A*_ Receptor

**DOI:** 10.1371/journal.pone.0168964

**Published:** 2017-01-13

**Authors:** Guiyun Mi, Shuai Liu, Jian Zhang, Huichun Liang, Yunyun Gao, Nuomin Li, Boyang Yu, Hongju Yang, Zheng Yang

**Affiliations:** 1 Beijing Institute of Basic Medical Sciences, Haidian District, Beijing, China; 2 Department of Complex Prescription of Traditional Chinese Medicine, China Pharmaceutical University, Nanjing, China; 3 The 89 Hospital of PLA, WeiFang, China; Xi'an Jiaotong University School of Medicine, CHINA

## Abstract

Tetrahydroprotoberberines (THPBs) are isoquinoline alkaloids isolated from the Chinese herb *Corydalis yanhusuo*. In the present study, we performed competitive binding assays to examine the binding of *l*-THBr to neurotransmitter receptors known to be involved in sedation, hypnosis and anxiety. Our results show that *l*-THBr does not interact with GABAergic receptors but has binding affinities for dopamine and serotonin receptors. In addition, cAMP and [^35^S]GTPγS assays were used to determine the agonist or antagonist properties of *l*-THBr at dopamine (D_1_, D_2_) or serotonin (5-HT) receptors. Our results show that *l*-THBr displays D_1_ and D_2_ antagonist and 5-HT_1A_ agonist properties. Moreover, *l*-THBr-treated rodents exhibit anxiolytic-like effects in the light/dark box and elevated plus-maze tests, and the anxiolytic effect of *l*-THBr can be reduced by WAY-100635, a selective 5-HT_1A_ receptor antagonist. Our results suggest that *l*-THBr may produce potent anxiolytic-like effects mainly through serotonin receptors.

## Introduction

Anxiety is a common mental state provoked in anticipation of a threat or potential threat, which may become an illness when excessive or inappropriate [1, 2]. The major physical and mental symptoms of anxiety include racing thoughts, nervousness, tremor, insomnia, emotional discomfort and agitation [3, 4]. As one of the most common psychiatric illnesses, anxiety disorders cause a prominent health care problem worldwide.

For over a century, researchers have searched for effective and safe agents to treat anxiety disorders. Benzodiazepines have been the mainstay of treatment since chlordiazepoxide was introduced in 1960 [5]. However, their therapeutic efficacy is limited due to unwanted side effects such as sedation, muscle relaxation, retrograde amnesia [6, 7] and dependency liability [8]. Another class of drugs, partial agonists of the serotonergic 5-HT_1A_ receptor, such as buspirone, gepirone, and ipsapirone, was identified as valuable for improving the clinical management of anxiety [9], but their therapeutic effects are delayed for 1–3 weeks [10]. Therefore, there is a demand for robust anxiolytic compounds that have fewer side effects and a more immediate onset of action.

**Fig 1:**
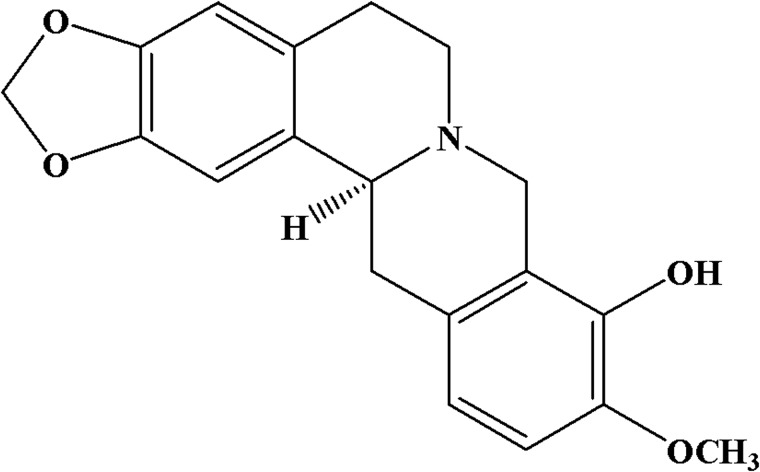
Chemical structure of levo-tetrahydroberberrubine (*l*-THBr).

Tetrahydroprotoberberines (THPBs) are isoquinoline alkaloids isolated from the Chinese herb *Corydalis yanhusuo*. *l*-Tetrahydropalmatine (*l*-THP), the main active ingredient of *C*. *yanhusuo*, has been used for more than 40 years in China as a treatment for chronic pain and anxious insomnia [11,12]. *l*-THP displays D_1_ and D_2_ antagonist properties and shows anti-addictive effects in animal models [13–16]. In addition, *l*-stepholidine (*l*-SPD), another derivative of tetrahydroprotoberberines, displays D_1_ agonist and D_2_ antagonist effects [17]. *l*-SPD has attracted much attention for its potential efficacy as a schizophrenia treatment [18–20]. However, *l*-SPD’s poor bioavailability and high industrial production cost limits its use [21]. Therefore, a new derivative of THPBs, levo-tetrahydroberberrubine (*l*-THBr) (Fig 1) was synthesized. In the present study, we examined the binding features of *l*-THBr to neurotransmitter receptors using competitive binding assays to address possible interactions with these receptors. Moreover, we characterized the functional activity of *l*-THBr at cloned D_1_ and D_2_ dopamine receptors and rat hippocampal 5-HT_1A_ serotonin receptors. In addition, the anxiolytic-like effects of *l*-THBr in two experimental animal models of anxiety were evaluated.

## Materials and Methods

### Animals

The experimental procedures were approved by the Beijing Institute of Basic Medical Science Institutional Committee on Animal Care and Use, and all efforts were made to minimize animal suffering and reduce the number of animals used for experiments. Male Sprague-Dawley (SD) rats and male CD-1 ICR mice (body mass of 18–22 grams) were purchased from Vitalriver Experimental Animal Center (Beijing, China). All animals were maintained under standard laboratory conditions and kept in temperature- and humidity-controlled rooms (21–22°C, 50%-60% humidity) on a 12 hour light-dark cycle (lights on from 7:00 am to 7:00 pm). All mice were used only once, and all behavioral experiments were performed between 8:00 and 12:00 am.

### Reagents and drug treatments

Quinpirole, buspirone, 8-OH-DPAT, SCH 23390, [^35^S]GTPγS and GTPγS were purchased from Sigma (St. Louis, MO, USA). SKF 38393 was purchased from Tocris (Bristol, United Kingdom). The cAMP Assay Kit was purchased from CISBIO (Catalog Number: 62AM4PEC). WAY100635 was obtained from Selleck (Texas, USA) and was dissolved in 0.9% saline. *l*-THBr was synthesized by the Department of Complex Prescription of Traditional Chinese Medicine (TCM), China Pharmaceutical University. It was dissolved in 0.1 mol/L H_2_SO_4_, diluted with sterile water and adjusted to pH 5–6 with 0.1 mol/L NaOH. The vehicle was prepared as above without drug. Diazepam (DZP) was purchased from Tianjin Jinyao Amino Acid Co., Ltd. (Tianjin, China) and was dissolved with control vehicle. For in vitro assays, all compounds were dissolved in DMSO and diluted with a solution of HBSS plus 20 mM HEPES.

### Radio-ligand binding assay

To determine the possible targets of *l*-THBr action, the binding affinities of *l*-THBr to neurotransmitter receptors known to be involved in sedation, hypnosis and anxiety were investigated. Radio-ligand binding assays were performed by Caliper Lifescience (Hopkinton, MA, USA). The screening was carried out at a concentration of 10 μM *l*–THBr to test its ability to inhibit the binding of radioligands to their corresponding receptors. The results were expressed as percentage of inhibition of labeled ligand binding to individual receptors. Significant binding activity was defined as ≥50%.

### cAMP assay for binding properties at D_1_ receptor

Dopamine receptors can be categorized into two classes: Gαs protein coupled receptors (D_1_ and D_5_), or Gαi protein coupled receptors (D_2_, D_3_, and D_4_). Activation of D_1_ receptors can excite adenylate cyclase activity and increase cyclic adenosine monophosphate (cAMP). cAMP assays were used to determine the agonist or antagonist properties of *l*-THBr at the dopamine D_1_ receptor.

CHO K1 cells stably expressing D_1_ receptors were purchased from Genscript (Catalogue Number: M00247, Gene Number NM_000794). The cells were seeded in Ham’s F12 containing 10% fetal bovine serum and 200 μg/ml zeocin. On the day of the assay, 5 μl of cell suspension (3000 cells) was seeded on a 384-well plate. The assay was performed according to the manufacturer’s instructions. To test for agonist effects at the D1 receptor, the compounds (a known D_1_ agonist SCH38393 or *l*-THBr) were added from stocks two-fold more concentrated than the final concentration. In another experiment to test for antagonist effects, the known D_1_ receptor antagonist SCH23390 or *l*-THBr was added to the system in the presence of the D_1_ receptor agonist SCH38393 (10 μM). After incubation (30 min at room temperature), 10 μL of HTRF reagents (cAMP-XL665 and anti-cAMP cryptate) were added. The signal was quantified after one hour of incubation at room temperature. The fluorescence intensity ratio (A_665nm_/A_620nm_ x10^4^) was calculated.

### [^35^S]GTPγS assay for binding properties at D_2_ and 5-HT_1A_ receptors

D_2_-D_4_ dopamine receptors and 5-HT_1A_ serotonin receptors are Gαi-coupled receptors that mediate inhibitory neurotransmission. We conducted [^35^S]GTPγS assays to determine the agonist or antagonist properties of *l*-THBr at D_2_ dopamine receptors or 5-HT_1A_ serotonin receptors. HEK293 cells stably expressing D_2_ receptors were provided by the Beijing Institute of Pharmacology and Toxicology (Beijing, China). The membrane preparation of D_2_-expressing HEK293 cells and rat hippocampal tissues highly expressing 5-HT_1A_ receptors were prepared as previously described [22–23]. Briefly, 10 SD rats were anesthetized and decapitated, and the hippocampi were quickly dissected and stored at -80°C until use. D_2_ receptor-expressing HEK293 cells and the hippocampal tissue were each homogenized at 4°C in 50 mM Tris-HCl buffer (pH 7.4). The homogenates were centrifuged at 2500 × g for 6 min, and the supernatant was further centrifuged for 20 min at 40000 × g. Membranes were re-suspended in Tris-HCl buffer and stored at -80°C until use.

[^35^S]GTPγS assays were performed in a total volume of 0.5 ml at 4°C. The incubation mixtures were prepared in glass tubes and consisted of membrane preparations (20 μg of protein), GDP (15 μM) and [^35^S]GTPγS (0.2 nM). Nonspecific binding was determined in the presence of unlabeled GTPγS (40 μM) following a 60 min incubation period at 30°C in the absence or in the presence of different concentrations of drugs (*l*–THBr, quinpirole and 8-OH-DPAT: 10^-10^–10^-5^ M; or quinpirole: 10 μM). The reactions were stopped with ice-cold Tris-HCl buffer and rapidly filtered through Whatman GF/B filters. Filters were quickly washed five times with 3 ml ice-old Tris-HCl and placed in scintillation cocktail solution. Bound radioactivity was determined by liquid scintillation counting. Drug effects were expressed as drug-induced increase in binding over basal binding (binding in the absence of drugs). Curves were fitted by non-linear regression analysis to the equation *Y = Bottom + (Top—Bottom) / {1 + 10 ^ ((lg*^*EC5−X*^) * Hill Slope)}, where Top and Bottom are plateaus in the units of the Y axis, EC_50_ is the concentration of agonist that gives a response halfway between Bottom and Top, and Hill Slope describes the steepness of the family of curves. The inhibitory effect of *l*–THBr was determined by a similar equation to obtain the IC_50_ values.

### Locomotor activity test

The spontaneous activity video analysis system consists of 8 sound-attenuated chambers (40 cm × 40 cm × 65 cm) with a built-in infrared camera. (JL Behave, Shanghai Ji-Liang Software Technology Co., Ltd). During the behavioral tests, the experimenter was outside the testing room, and the chambers were cleaned between successive runs. Forty male CD-1 ICR mice were allowed to acclimate for three days in home cages and were handled for another three days to minimize stress after arrival in the animal facility. On the following day, after habituation to the activity chambers for 60 min, mice were administered vehicle, diazepam (DZP, 2 mg/kg, i.p.) or *l*–THBr (1, 5, or 10 mg/kg, i.p.) and immediately placed into the test chambers to record their locomotor activity for 60 min.

### Light-dark box test

The light-dark box test is a sensitive model to detect activity in disorders related to anxiety, based on the innate aversion of rodents to brightly lit areas and on their spontaneous exploratory behavior in response to a novel environment [24]. The light-dark transition box is a polypropylene animal cage (44 cm × 21 cm × 21 cm), which is divided into two compartments, a light box (illuminated by a 60 W light source with 1000 lx light intensity) and a dark box. Forty-eight male CD-1 ICR mice were placed in the light box 30 min after *l*–THBr or DZP injection and allowed to move freely to both boxes for 5 min. The number of transitions between the two boxes were recorded by a video camera.

### Elevated plus-maze test

The elevated plus-maze test is widely used for the screening and evaluation of anxiolytic drugs [25–26]. The apparatus consists of two open arms (30 cm × 5 cm) and two enclosed arms (30 cm × 5 cm × 15 cm), which is elevated 45 cm above the ground. The entire maze was made of clear Plexiglas and illuminated by four 30 W white lights with 300 lx light intensity arranged as a cross 100 cm above the maze. 48 male CD-1 ICR mice were randomly divided into either the vehicle group, one of three doses of *l*–THBr groups, or the diazepam group. 30 min after injection, mice were gently placed on the center platform facing an open arm, and the number of entries and the time spent in both arms were recorded by a video camera for 5 min. The results were expressed as the percentage of entries into the open arms (%) = (the number of entries into the open arms / the total number of entries into the four arms) × 100%; and the percentage of time spent in the open arms (%) = (time spent in the open arms / total time spent in the four arms) × 100%.

In a separate experiment, WAY100635 (a 5-HT_1A_ antagonist) was used to test whether the anxiolytic-like activity of *l*-THBr is mediated by the activation of the 5-HT_1A_ receptor. Forty male CD-1 ICR mice were randomly divided into one of four groups: vehicle, WAY100635 (3 mg/kg, i.p.), *l*–THBr (5 mg/kg, i.p.) or WAY100635 (3 mg/kg, i.p.) + *l*–THBr (5 mg/kg, i.p.). Mice were administered vehicle or WAY100635 followed by vehicle or *l*–THBr (5 mg/kg) injections 15 min later. The test was performed 30 min after the administration of *l*-THBr or vehicle.

### Statistical analysis

All data sets were initially checked for normality and homogeneity of variance. The data were expressed as the mean ± S.E.M and assessed using one-way ANOVA followed by Bonferroni post hoc comparisons. A 2 x 2 factorial ANOVA was used to determine interaction effects for WAY100635 and *l*-THBr. *P* < 0.05 was defined as a statistically significant difference.

## Results

### Binding affinity of *l*-THBr to neurotransmitter receptors

The in vitro receptor competitive binding data illustrate that *l*-THBr at a concentration of 10 μ M has a high binding affinity for D_1_, D_2_, and D_3_ dopamine and 5-HT_1A_ serotonin receptors but not for GABA or glutamate receptors. The inhibition of ligand binding to D_1_, D_2_, and D_3_ dopamine and serotonin 5-HT_1A_ receptors was 100.6%, 98.41%, 70.63% and 79.3%, respectively (Table 1).

**Table 1:** The Inhibition of ligand binding to neurotransmitter receptors by *l*-THBr.

Receptor	Radioligand	Reference compound	Average inhibition percentage	Activity
Dopamine Transporter	[3H]WIN 35,428	GBR12909	8.8%	No
Dopamine, D1 (h)	[3H]-SCH23390	SCH23390	100.61%	Yes
Dopamine, D2s (h)	[3H]-Raclopride	Haloperidol	96.43%	Yes
Dopamine, D3	[3H]7-OH-DAPT	(+/-)-7-OH-DAPT HBr	77.61%	Yes
Dopamine, D4.4 (h)	[3H]-YM-09151-2	Haloperidol	79.55%	Yes
GABA A, Agonist Site	[3H]GABA	GABA	7.89%	No
GABA A, BDZ, Alpha 1 site	[3H]Flunitrazepam	Ro5-1788	-3.30%	No
GABA-B	[3H]CGP 54626A	(+/-) Baclofen	-6.94%	No
Glutamate, AMPA Site (Ionotropic)	[3H]AMPA	(+/-) AMPA HBr	0.31%	No
Glutamate, Kainate Site (Ionotropic)	[3H]Kainic acid	Kainic Acid	-0.89%	No
Glutamate, MK-801 (Ionotropic)	[3H] MK-801	(+)-MK-801 HMaleate	6.41%	No
Glutamate, NMDA Agonist Site (Ionotropic)	[3H]CGP 39653	NMDA	4.79%	No
Glutamate, NMDA, Phencyclidine Site (Ionotropic)	[3H]TCP	(+)-MK-801 Hydrogen	-12.74%	No
Glutamate, NMDA, Glycine (stry-insens Site (Ionotropic)	[3H]-MDL-105,519	MDL-105,519	1.79%	No
Glycline, Strychnine-sensitive	[3H]Strchnine	Strychnine nitrate	21.26%	No
Serotonin Transporter	[3H]Citalopram,N-Methyl	Imipramine HCl	34.43%	No
Serotonin, 5HT1A (h)	[3H]-8-OH-DPAT	8-OH-DPAT	78.31%	Yes
Serotonin, 5HT1D	[3H]5-CT	5-CT	42.28%	No
Serotonin, 5HT2A	[3H]Ketanserin	Methysergide maleate	40.90%	No
Serotonin, 5HT2C	[3H]Mesulergine	Mianserin	30.65%	No
Serotonin, 5HT3	[3H]GR 65630	MDL 72222	23.02%	No
Serotonin, 5HT4	[3H] 113808	Serotonin	32.84%	No
Serotonin, 5HT5A (h)	[3H]-LSD	Methiothepin mesylate	23.79%	No
Serotonin, 5HT6 (h)	[3H]-LSD	Methiothepin mesylate	22.79%	No
Serotonin, 5HT7 (h)	[3H]LSD	5-CT	54.82%	Yes

Significant binding affinity of *l*-THBr was defined as greater than 50% inhibition of ligand binding. The concentration of *l*-THBr and ligands was 10 μmol/L. Each value was determined by two independent experiments.

### cAMP assay for D_1_ receptor activity

**Fig 2:**
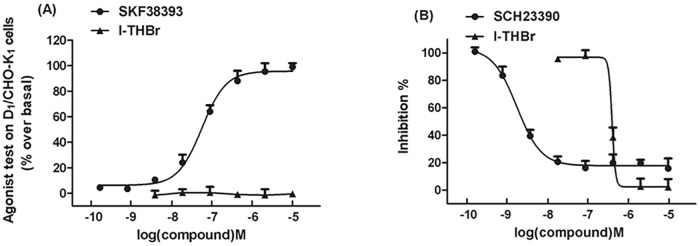
*l*-THBr acts as an antagonist at D_1_ dopamine receptors. (A) The effects of the D_1_ receptor agonist SKF 38393 and *l*-THBr on cAMP formation in CHO cells expressing the D_1_ receptor. The EC_50_ of SKF 38393 was calculated. (B) The inhibition of cAMP formation induced by SKF 38393 (10 μM) by the D_1_ receptor antagonist SKF 23390 and *l*-THBr in CHO cells expressing the D_1_ receptor. Curves were fitted by non-linear regression analysis. The half maximal inhibitory concentration (IC_50_) was calculated. The means ± S.E.M. from three independent experiments performed in duplicate are shown.

As shown in Fig 2A, the D_1_ receptor agonist SKF 38393 but not *l*-THBr induced a dose-dependent increase in cAMP production in CHO cells stably expressing the D_1_ receptor, with an EC_50_ of 49.1 nM. In contrast, the D_1_ receptor antagonist SCH23390 inhibited the production of cAMP induced by SKF 38393 (10 μM) in a dose-dependent manner with an IC_50_ of 1.42 nM. *l*-THBr also inhibited cAMP production with an IC_50_ of 361 nM (Fig 2B), indicating that *l*-THBr is a D_1_ receptor antagonist. The maximum inhibition by *l*-THBr is 98.75% ±3.49.

### [^35^S]GTPγS assay for D_2_ receptor activity

**Fig 3:**
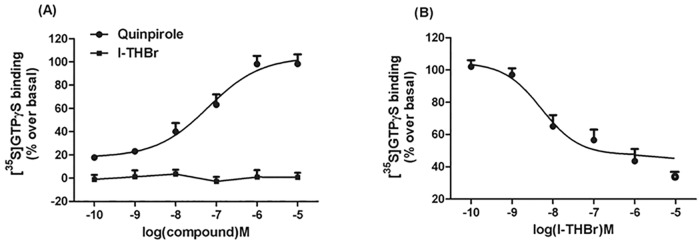
*l*-THBr acts as an antagonist at D_2_ dopamine receptors. (A) Dose-response curves of quinpirole- or l-THBr–induced [^35^S]GTPγS binding in HEK293 cells expressing the human D_2_ dopamine receptor. (B) *l*-THBr significantly attenuates the binding of [^35^S]GTPγS to D_2_ receptors induced by quinpirole (10 μM). Curves were fitted by non-linear regression analysis. The means ± S.E.M from three independent experiments in duplicate are shown.

As shown in Fig 3A, the D_2_ receptor agonist quinpirole but not *l*-THBr induced a dose-dependent increase in [^35^S]GTPγS binding in HEK293 cells expressing the D_2_ receptor, with an EC_50_ of 63.71 nM. *l*-THBr significantly attenuates the effect of 10 μM quinpirole on [^35^S]GTPγS binding to D_2_ receptors in a concentration-dependent manner with an IC_50_ of 5.264 nM, indicating that *l*-THBr is a D_2_ receptor antagonist (Fig 3B).

### [^35^S]GTPγS assay for 5-HT_1A_ receptor activity

**Fig 4:**
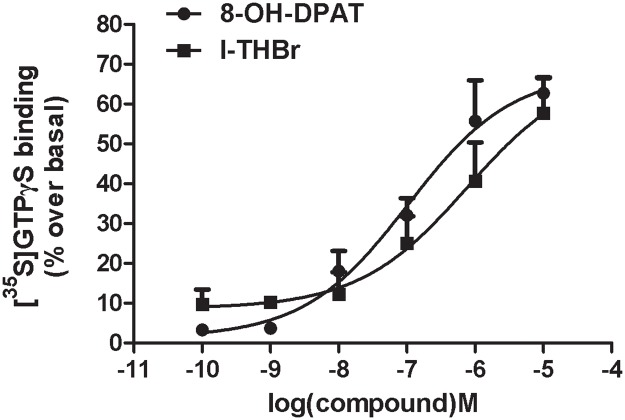
*l*-THBr acts as an agonist in rat hippocampal 5-HT_1A_ receptors. *l*-THBr and the 5-HT_1A_ agonist 8-OH-DPAT increase [^35^S]GTPγS binding to 5-HT_1A_ receptors in rat hippocampus in a dose-dependent manner. Curves were fitted by non-linear regression analysis. The means ± S.E.M. from at least three independent experiments in duplicate are shown.

As shown in Fig 4, both *l*-THBr and the 5-HT_1A_ agonist 8-OH-DPAT increased [^35^S]GTPγS binding to 5-HT_1A_ receptors in rat hippocampus in a dose-dependent manner, with an EC_50_ of 234.7 nM for *l*-THBr 98.2 nM for 8-OH-DPAT, indicating that *l*-THBr is an agonist of the 5-HT_1A_ receptor.

### The effects of *l*–THBr on spontaneous locomotor activity

**Fig 5:**
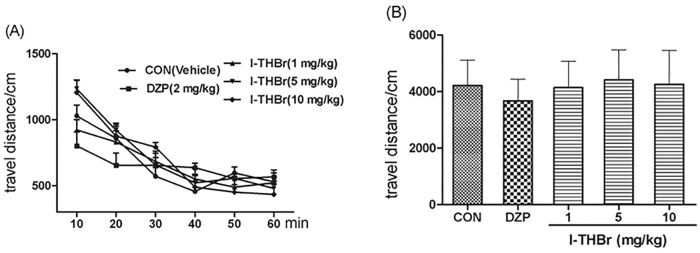
Effects of *l*-THBr on locomotor activity in mice. (A) The distance traveled within a 10 min interval. (B) The total distance traveled within 60 min. The data are represented as the means ± S.E.M.

Mice were administered *l*–THBr (1, 5, or 10 mg/kg, i.p.), diazepam (DZP 2 mg/kg, i.p.) or vehicle and immediately placed into locomotor chambers to test their locomotor activity for 60 min. Travel distance (in cm) was calculated every 10 min. Diazepam or *l*–THBr administration had no influence on locomotor activity at the test doses (F_(3, 35)_ = 1.914, P > 0.05). The results are shown in Fig 5.

### The effects of *l*-THBr in the light/dark box test

**Fig 6:**
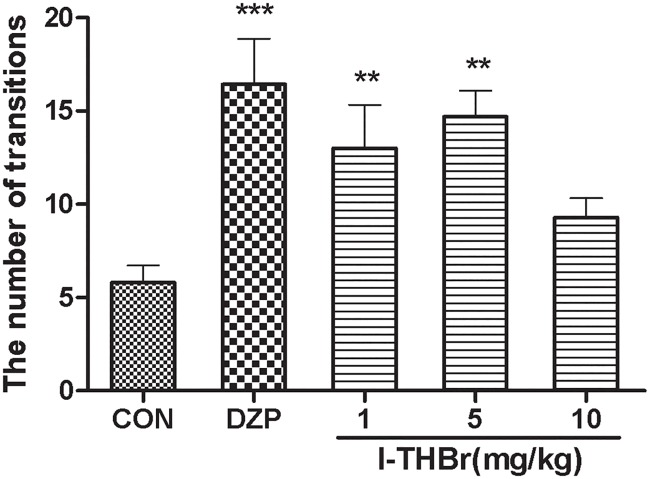
Effects of *l*-THBr on the number of transitions in the light/dark box test. *l*-THBr (1, 5 or 10 mg/kg i. p.), DZP (2 mg/kg), or vehicle was administered 30 min before the test. Administration of DZP (2 mg/kg) or *l*-THBr (1 or 5 mg/kg) increased the number of transitions between the light/dark sides (*P* < 0.05 compared with the vehicle group). The data are represented as the means ± S.E.M. *P < 0.05, **P < 0.01, compared with the vehicle group.

One-way ANOVA revealed significant differences among treatment groups (F_(4, 43)_ = 6.068, *P*<0.001). The administration of DZP (2 mg/kg) or *l*-THBr (1 or 5 mg/kg) increased the number of transitions between the light/dark sides (*P* < 0.05 compared with the vehicle group). The results are shown in Fig 6.

### The effects of *l*–THBr in the elevated plus maze

**Fig 7:**
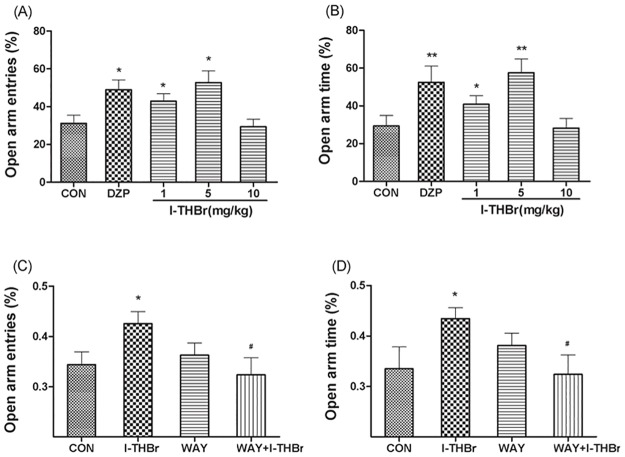
The effects of *l*-THBr on the elevated plus maze task. (A) Effects of diazepam (DZP) or *l*-THP on the percentage of entries into open arms. (B) Effects of diazepam (DZP) or *l*-THP on the percentage of time spent in the open arms during a 5 min period. (C) The anxiolytic-like effects of *l*–THBr were reversed by co-administration of WAY100635, a 5-HT_1A_ receptor antagonist. The data were expressed as the percentage of entries into the open arms. (D) The anxiolytic-like effects of *l*–THBr were reversed by co-administration of WAY100635. The data were expressed as the percentage of the time spent in the open arms. In the WAY100635 antagonist groups, mice were administered saline or WAY100635 (3 mg/kg, i.p.) followed by administration of a vehicle or *l*-THBr (5 mg/kg, i.p.) 15 min later. The test was performed 30 min after the administration of *l*-THBr or vehicle. The data are represented as the means ± S.E.M. *P < 0.05, **P < 0.01, compared with the vehicle group. ^#^*P* < 0.05, compared with the *l*-THBr group.

A one-way ANOVA revealed a significant difference among the treatment groups in the percentage of entries into the open arms (F_(4, 43)_ = 6.141, *P* < 0.001) and the percentage of time spent in the open arms (F_(4, 43)_ = 5.557, *P* < 0.01). DZP (2 mg/kg, i.p.) produced a significant increase in the percentage of arm entries and the percentage of time spent in the open arms (*P* < 0.05 compared with the control group), indicating the predictive validity of the elevated plus maze model. *l*–THBr (1 or 5 mg/ kg, i.p.) increased the ratio of entries into the open arms and the ratio of time spent in the open arms (*P* < 0.05 compared with the control group), indicating that *l*–THBr has anxiolytic effects in this animal model. The results are shown in Fig 7A and 7B.

Fig 7C and 7D show the effects of WAY-100635, a 5-HT_1A_ receptor antagonist, on the anxiolytic effects of *l*-THBr. A 2×2 factorial ANOVA revealed a significant interaction between WAY-100635 (3 mg/kg, i.p.) and *l*-THBr (5 mg/kg, i.p.) (for percentage of open arm entries: F_(1,33)_ = 6.33, P < 0.05; for percentage of open arm time: F_(1,33)_ = 6.38, P < 0.05). Subsequent analysis of single treatment effects indicated that there was a difference between the control and *l*-THBr-treated groups (for percentage of open arm entries: F_(1, 33)_ = 6.22, P < 0.05; for percentage of open arm time: F_(1, 33)_ = 5.94, P < 0.05). The analysis also indicated a significant difference between the *l*-THBr (5 mg/kg) and WAY100635 + *l*-THBr groups (for percentage of open arm entries: F_(1, 33)_ = 8.95, P < 0.01; for percentage of open arm time: F_(1, 33)_ = 7.12, P < 0.05). These results show that the anxiolytic-like effects of *l*–THBr were significantly reversed by co-administration of the 5-HT_1A_ antagonist WAY100635 (3 mg/kg, i. p.).

## Discussion

In the present study, we evaluated the anxiolytic-like effects of *l*-THBr in behavioral models of anxiety. We found that intraperitoneal administration of *l*-THBr produced anxiolytic-like effects in the elevated plus maze and light-dark box tests. In addition, *l*-THBr had a high affinity for D_1_, D_2_-like dopamine and serotonin 5-HT_1A_ receptors and exhibited D_1_, D_2_ antagonist and 5-HT_1A_ agonist properties.

The anxiolytic mechanism of diazepam occurs mainly through benzodiazepine receptors, which are present in the GABA receptor pentameric complex. Thus, diazepam induces sedative effects by increasing the opening frequency of the associated chloride ion channel and hyperpolarizing the membrane [27]. In the present study, diazepam showed a significant and stable anxiolytic-like effect in the male ICR mice, consistent with some previous studies [28]. The in vitro receptor competitive test results demonstrated that *l*-THBr does not interact with inhibitory GABAergic receptors at benzodiazepine (BDZ) sites but mainly binds to dopamine and serotonin receptors. Thus, *l*-THBr works well at relieving anxiety without causing sedative effects.

Anxiety disorders are associated with the dysfunction of a number of neurotransmitters and their receptors, including dopamine and serotonin [29–33]. An increase in dopaminergic transmission has been demonstrated to aggravate anxiety [34], and the D_1_ receptor antagonist SCH23390 exhibits clear anxiolytic-like effects [33, 35, 36]. However, D_2_ receptor ligands can produce either anxiogenic [33, 37] or anxiolytic-like effects in animal models [28, 38, 39]. D_1_ dopamine receptors are mainly found at postsynaptic sites, whereas D_2_ dopamine receptors are localized both presynaptically (where they act as autoreceptors) and postsynaptically. Therefore, D_2_ antagonists may block presynaptic D_2_ dopamine autoreceptors and increase the release of dopamine, which in turn modulate anxiety-like behaviors by acting on postsynaptic D_2_ dopamine receptors [40–41]. Whether D_2_ antagonists exert effects through presynaptic D_2_ receptor or postsynaptic D_2_ receptors may largely depend on the test doses used [39–42]. However, in our studies, the anxiolytic-like effect of *l*-THBr in the elevated plus maze test was blocked by the 5-HT_1A_ antagonist WAY100635. Thus, our results suggest that the anxiolytic-like effects of *l*-THBr are probably mediated through a 5-HT_1A_ receptor mechanism.

Previous studies indicate that injection of 5-HT into the brain stem produces anxiety [43]. Moreover, the anxiolytic activities of 5-HT_1A_ full or partial agonists are thought to be the result of decreased 5-HT outflow and a reduction of serotonergic neuron activity via the activation of 5-HT_1A_ autoreceptors at presynaptic sites [44].

In addition, the interaction of D_2_ receptors and 5-HT_1A_ receptors plays an important role in mental disorders [45]. For example, the 5-HT_1A_ agonist buspirone at low doses of 1.25–5.0 mg/kg (which are relevant doses for the anxiolytic effects of buspirone) blocks presynaptic D_2_ autoreceptors [46, 47]. In addition, aripiprazole or SSR181507 (a combined D_2_ antagonist and a 5-HT_1A_ partial agonist, respectively) improve depression and anxiety symptoms in patients with schizophrenia [48, 49]. Based on these findings, it has been proposed that a combination of D_2_ antagonistic and 5-HT_1A_ agonistic properties would offer additional advantages in treating some mental disorders, such as anxiety, depression (for fast onset anti-depressants) and schizophrenia [50, 51].

In conclusion, *l*-THBr exhibits anxiolytic activity in two animal models of anxiety. Activation of 5-HT_1A_ autoreceptors and a decrease in serotonergic activity most likely contributes to the anxiolytic activity of *l*-THBr in these tests. The ability of *l*-THBr to exert effective anxiolytic activity without sedative effects suggests a potential use for *l*-THBr as a superior treatment for anxiety.
